# Type 1 tympanoplasty in pediatric patients: a review of 102 cases

**DOI:** 10.1186/s12887-018-1326-1

**Published:** 2018-11-06

**Authors:** Deniz Baklaci, Ismail Guler, Ihsan Kuzucu, Rauf Oguzhan Kum, Muge Ozcan

**Affiliations:** Numune Training and Research Hospital, Department of Otolaryngology, 931st Avenue 945st Street No:5/3 Çankaya, Ankara, Turkey

**Keywords:** Type 1 tympanoplasty, Children, Prognostic factors, Graft success, Hearing

## Abstract

**Background:**

The aim of this study was to investigate the success of type 1 tympanoplasty in pediatric patients with chronic otitis media, and to evaluate the prognostic factors that may influence its success.

**Materials and methods:**

Medical records of 102 children aged between 8 and 18 years (46 female, 56 male) who underwent type 1 tympanoplasty for chronic tympanic membrane perforation between January 2010 and July 2017 were reviewed. Age, gender, condition of contralateral ear (unilateral, bilateral), type (central, marginal) and location of perforation (anterior, posterior, inferior), graft material (fascia, cartilage), pre- and post-operative hearing levels, mean air-bone gap (ABG), surgical approach (postauricular, endaural) and length of follow up were recorded.

**Results:**

Anatomical and functional success (ABG < 20 dBHL) rates were 86.3% (88 patients) and 74.5% (76 patients) after a mean follow-up of 32 ± 16.55 months, respectively. The mean hearing improvement was 10.77 ± 10.45 dBHL. The graft success rates were significantly higher in tragal cartilage group (95.1%) than in temporalis fascia group (80.3%) (*p* = 0.033). Graft success was negatively affected by contralateral perforation (*p* = 0.003). All patients with bilateral perforations and graft failure were in temporalis fascia group. Age, type and location of perforation and surgical approach did not influence graft success (*p* > 0.05).

**Conclusion:**

Our results showed that type 1 tympanoplasty can be performed effectively in pediatric population regardless of age, location and type of perforation and surgical approach. Bilateral perforations are prone to reperforation, and should be treated with cartilage graft.

## Background

Tympanoplasty was popularized by Zollner and Wullstein [1] in the middle of 1950s. Today, the success rate of tympanoplasty still remains a considerable interest. There has been a particular attention to the outcomes of tympanoplasty in the pediatric age group since 1960s [2, 3]. In the literature, a success rate of tympanoplasties in children ranging from 35 to 94% [4, 5]. Numerous factors have been proposed for the reason of poorer tympanoplasty outcomes in children than in adults: higher frequency of upper respiratory infection and otitis media, poorer eustachian tube function and presence of hypertrophic adenoid tissue, and immaturity of the immune system [6–10]. Therefore, some authors do not propose tympanoplasty in pediatric age [10, 11]. They advocate that eustachian tube dysfunction results in inadequate ventilation to the middle ear, leading to the tympanic membrane (TM) perforation [7–10]. These results mitigating against delaying tympanoplasty in pediatric patients allows for the spontaneous closure of TM perforations. Another problem in pediatric tympanoplasty is thata narrow external auditory canal may complicate the surgery in some patients while postoperative dressings and care may be compelling and unpleasant, especially in uncooperative children.

Nevertheless, studies showed that tympanoplasty success rates in pediatric population were similar to adults [6, 12, 13]. The philosophy of performing tympanoplasty on the pediatric population is based on facts that cannot be disregarded. The presence of hearing loss in childhood has negative effects on the development of speech, language and learning skills. It has been showed that children with hearing loss have some difficulties with reaching their full academic potential [14]. Since children have an excellent cochlear reserve, they are more likely to benefit from tympanoplasty in terms of hearing improvement. On the other hand, the risk for a long-standing perforation isthat the squamous epithelium may enter the middle ear through the perforation and this may result in cholesteatoma formation in the middle ear cavity [15]. Therefore, closure of TM perforation prevents cholesteatoma formation and ossicular damage in the long-term. A successful tympanoplasty provides children a safe, non-draining and care-free ear and also the ability to participate in water activities and other social activities without restriction.

There are various factors that may affect the surgical success of tympanoplasty in children, such as age, the size and location of perforation, previous otological operations (ventilation tube, myringotomy, tympanoplasty), condition of the operated and contralateral ear (dry vs draining), presence of adenoid hypertrophy, function of the eustachian tube, choice of graft material, surgical technique and surgeon’s experience [8–10, 16]. However, there is still a lack of certainty on which factors contribute to a successful closure due to inconsistencies in demographic and surgical parameters have been evaluated in the literature.

In this study, we aimed to investigate the surgical and functional outcomes of type 1 tympanoplasty in pediatric population and determine the factors influencing the success of tympanoplasty.

## Methods

Medical records of 147 pediatric patients who underwent primary tympanoplasty in our clinic between January 2010 and July 2017 were reviewed. The casespresenting with wet ear, cholesteatoma, tympanosclerosis, ossicular chain erosion, combined with mastoidectomy or ossiculoplasty, and patients younger than 8 years old and older than 18 at the time of the surgery and whose first follow-ups were less than 12 months,were excluded from the study.

The study was approved by the local ethics committee (no: E-18-1907), and conducted in accordance with the ethical principles described by the Declaration of Helsinki. Prior written informed consent was obtained from the parents or guardians of the children who served as participants, and of participants 16 years or older, and assent form participants under 16 years of age.

For each patient we analyzed age, gender, condition of contralateral ear (unilateral/bilateral), type (central/marginal) and location of the perforation (anterior/posterior/inferior), graft material (temporal fascia/tragal cartilage), preoperative and postoperative hearing levels, surgical approach (postauricular/endaural), and length of follow-up. Patients were divided into two groups by age: ≤12 years and 13–18 years, as those that are thirteen years old and older are considered adolescents, while those twelve years old and younger are considered children [17].

Type of perforation was described as marginal in cases which the anulus was involved, otherwise it was described as central. Location of perforation categorized into three groups regarding its relation to handle of malleus: anterior, posterior and inferior.

The nasal cavity and nasopharynx were examined by nasal endoscopy. The patients with allergic rhinitis were treated with nasal steroids and antihistaminic drugs for at least two months, and the operation was performed following the evaluation of allergic rhinitis symptoms and nasal cavity examination after treatment. The patients with adenoid vejetation were treated with adenoidectomy, and the operation was performed after three months from adenoidectomy surgery.

All the children were operated witha microscope (Möller-Wedel Optical®; Hamburg, Germany) under general anesthesia. All tympanoplastieswere performed using the underlay technique with temporalis fascia or tragal cartilage through postauricular or endaural incision.

All the children underwent detailed preoperative and postoperative 6th month pure-tone audiometry (PTA) measurements at 0.5, 1, 2 and 4 kHz frequencies using an Interacoustic AC-40 (Middelfart, Denmark) clinical audiometer. Preoperative and postoperative air and bone conduction thresholds were measured for four frequencies. The air–bone gap (ABG) was calculated as the average difference between the air conduction and the bone conduction at 0.5, 1, 2, and 4 kHz. A successful functional result wasdefined as a postoperative ABG of 20 dB (dB) or less [18].

The postoperative follow-up controls take place generally at hospital at the 1st, 3rd, 6th, and 12th months, and annually there after. The surgical performance is measured by otoscopic and microscopic examinations 12 months after the surgery.

Statistical data analysis was performed using SPSS 21 (SPSS Inc., IBM company, Chicago). Data was expressed as mean ± standard deviation (S.D) and as proportion (%). Chi-square test was used for comprising of categorical data. Wilcoxon and Mann-Whitney U tests were used for non-parametric variables whereas independent and paired sample t test were used for parametric variables. The multivariate logistic regression analysis was used to identify independent predictors of functional success. A *p* value < 0.05 was accepted as statistically significant.

## Results

A total of 102 patients (46 female, 56 male) with 102 operated ears (66 left, 36 right) who met the criteria mentioned above were included in this study. The mean length of follow-up was 32 ± 16.55 months (range 12 to 66 months).

The prognostic factors regarding age, statue of contralateral ear, location and type of perforation, graft material and surgical approach are given in Table 1.

**Table 1:** Comparison of prognostic factors for surgical outcomes

	*n*	Graft success (*n*, %)	*p*
Younger group	28	24 (85.5)	
(≤12 ages)			N.S.^*^
Older group	74	64 (86.5)	
(13–17 ages)			
Unilateral perforation	73	68 (93.2)	0.003
Bilateral perforation	29	20 (69)	
Location of perforation			
Anterior	45	39 (86.7)	
Posterior	45	39 (86.7)	N.S.^*^
İnferior	12	10 (83.3)	
Type of perforation			
Central	77	68 (88.3)	N.S.^*^
Marjinal	25	20 (80)	
Graft material			
Fascia	61	49 (80.3)	0.033
Cartilage	41	39 (95.1)	
Approach			
Postauricular	78	67 (85.9)	N.S.^*^
Endaural	24	21 (87.5)	

N.S., ^*^p > 0.05

**Fig. 1:**
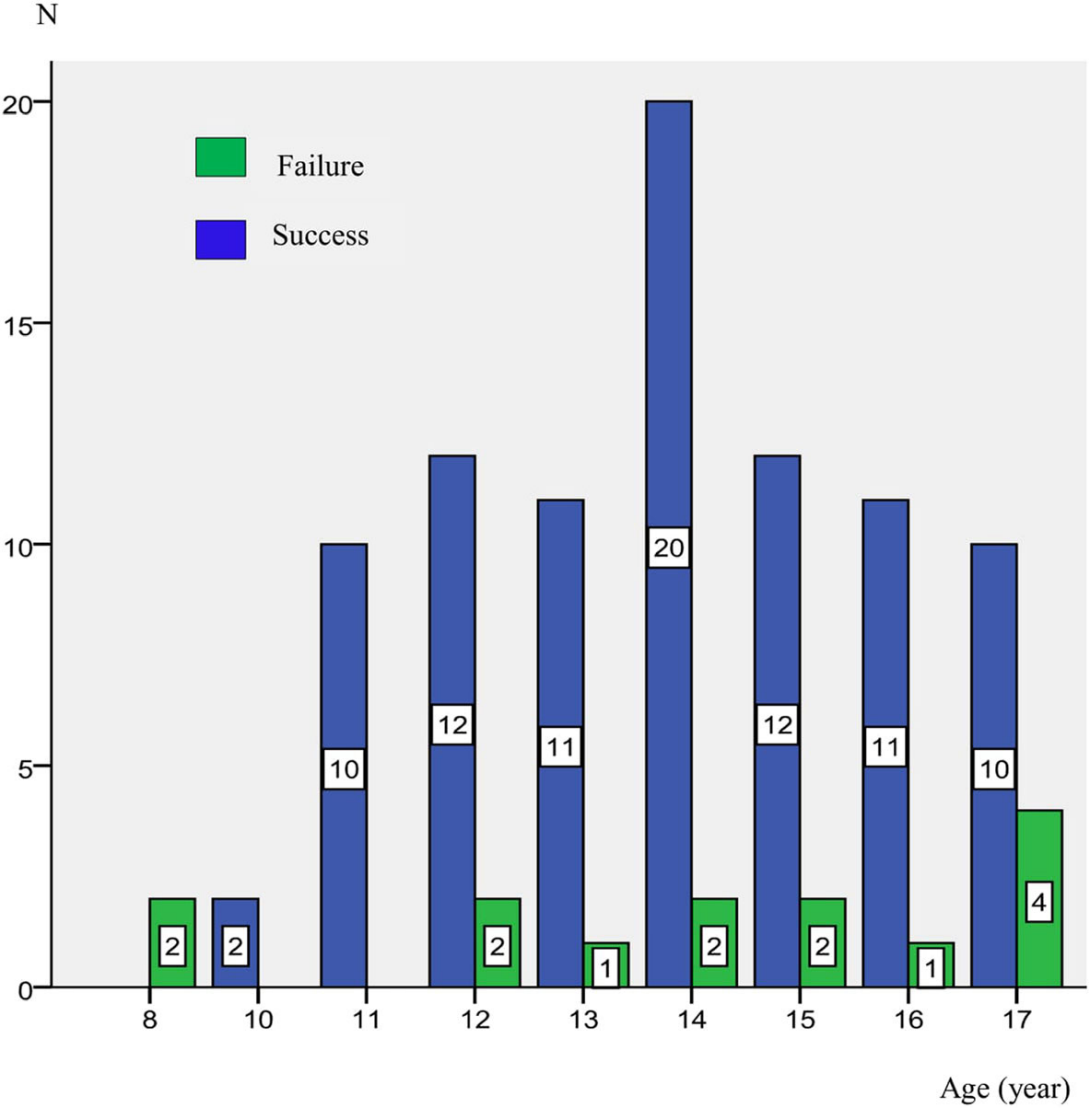
Tympanoplasty outcomes by age. Shows the distribution of graft take results of patients according to age

The mean age was 14 ± 2.10, ranging from 8 to 18 years old. There were 28 patients (27.4%) in the younger group and 74 patients (78.6%) in the older group. The mean ages were 11.21 ± 1.01 for 12 years old and younger and 14*.*92 ± 1.37 for 13 years old and older. Graft success rates were 85.5% (24 ears) for 12 years old and younger and 86.5% (64 ears) for 13 years old and older. There was no statistically significant difference in the graft success rates between age groups (*p* = 1.000). The distribution of tympanoplasty outcomes by age was given in Fig. 1.

The overall anatomical success rate of tympanoplasty was 86.3% (88 ears) at the last visit. During the follow-up period, recurrent perforation occurred in 13.7% of patients (14 ears) (Table 2).73 patients (71.6%) had unilateral perforation, 29 patients (28.4%) had bilateral perforation**.** Graft success rates were 93.2% (68 ears) in patients with unilateral perforations and 69.0% (20 ears) in patients with bilateral perforations, and the difference was statistically significant (*p* = 0.003).

**Table 2:** The anatomical and functional success

	*n*	%
Anatomical success	88	86.3
Functional success (< 20 dBHL ABG)	76	74.5

*ABG* air-bone gap

Of the 102 ears, 45 ears (44.1%) perforations were anterior, 45 (44.1%)were posterior, and 12 (11.8%) were inferior. Graft success rates were 86.7% (39 ears) in anterior perforations, 86.7% (39 ears) in posterior perforations, and 83.3% (10 ears) in inferior perforations. There was no statistically significant difference in the graft success rates between the location of perforations (*p* = 0.952).

Seventy seven ears (75.5%) were with a central perforation and 25 ears (24.5%) were marginal perforation. Graft success rate of central perforations was 88.3% (68 ears) and marginal perforations was 80.0% (20 ears). There was no statistically significant difference in the graft success rates between the type of perforations (*p* = 0.294).

Temporal fascia was used in 61 ears (59.8%) and tragal cartilage was used in 41 ears (40.2%). Graft success rate of fascia group was 80.3% (49 ears) and cartilage group was 95.1% (39 ears), and the difference was statistically significant (*p* = 0.033).

Seventy eight ears (76.5%) were operated via postauricular approach, 24 ears (23.5%) were operated via endaural. Graft success rate of postauricular group was 85.9% (67 ears) and endaural group was 87.5% (21 ears). There was no statistically significant difference in the graft success rates between the type of surgical approach (*p* = 0.573).

The mean preoperative and postoperative bone conduction thresholds were12.51 ± 8.18 dB and 9.74 ± 8.60 dB, respectively, and the difference was statistically significant (*p* < 0.001). The mean preoperative and postoperative air conduction thresholds were 39.36 ± 14.10 dB and 25.79 ± 15.57 dB, respectively, and the difference was statistically significant (*p* < 0.001). The mean preoperative and postoperative ABG were 26.85 ± 9.47 dB and 16.08 ± 10.46 dB, respectively, and the difference was statistically significant (*p* < 0.001) (Table 3). The mean hearing improvement was 10.77 ± 10.45 dB. The postoperative ABG was less than 20 dB in 76 patients, and the functional success rate was 74.5% (Table 2).

**Table 3:** Comparison of pre- and post-operative mean air-bone thresholds and ABG

	Pre-operative (dB ± S.D.)	Post-operative (dB ± S.D.)	*p*
Air conduction	39.36 ± 14.10	25.79 ± 15.57	< 0.001
Bone conduction	12.51 ± 8.1	9.74 ± 8.60	< 0.001
ABG	26.85 ± 9.47	16.08 ± 10.46	< 0.001

*dB* decibel, *ABG* air-bone gap, *S.D.* Standard deviation, *p* < 0.05

## Discussion

Success of pediatric tympanoplasty has been a topic of discussion for many years. In the literature, there are numerous studies that investigated it with quite variable results [4, 5, 10, 12]. The disparity of outcomes in pediatric tympanoplasty is attributed to different definition of success and heterogenity of the patients included. In the present study, the success rate of pediatric tympanoplasty was 86.3%, and this result is consistent with rates based on the literature. Factors such as age, location and type of perforation, surgical approach have no statistically significant effect on tympanoplasty outcomes. Status of contralateral ear and graft material influence the outcomes of tympanoplasty.

The timing of pediatric tympanoplasty is controversial. Improvement of eustachian tube function continues with aging children [19]. The reasons for the failure of the tympanoplasty results in early ages are thought to be inadequate function of the eustachian tube, weak immune system and recurrent upper respiratory tract infections [6, 8, 20]. There have been various suggestions by different authors regarding the age of the child before the surgery. Some recommended waiting until the ages of 6 [5], 7 [21] or 8 [9] for the operation while others observed more favorable results in children aged 9 [22], 10 [10] or 12 [8]. On the contrary, some authors declared that there was no correlation between age and surgical success [12, 22]. In a study [23], which included the surgical outcomes of tympanoplasty in 52 patients aged between 13 and 17 years old, the success rate was 80.8% similar to those found in adults. In our study, we compared two pediatric age groups (age 8–12 and age 13–18) in terms of tympanoplasty outcome, and found that tympanoplasty outcomes were favorable and similar in both groups (*p* > 0.05). Although a small number of patients were in the younger group, this is the first study to compare the success of tympanoplasty between adolescent and childhood groups.

Eustachian tube function is an important factor affecting the success of surgery [9, 17]. The status of contralateral ear can be a guide about eustachian tube dysfunction. Some authors [12] showed that the success rates of bilateral perforations decreased by half of unilateral perforations, and they proposed that high graft failures would be a result of severe eustachian dysfunction. In the present study, we used the status of contralateral ear as a predictor of eustachian tube function. There were five graft failures in unilateral group, and nine graft failures in bilateral group. Our results showed that the patients with unilateral perforation had higher graft success (93.2%) than patients with bilateral perforations (69%) (*p* = 0.003). Furthermore, we found that in all bilateral perforations that postoperative graft failure occurred, the temporal fascia had been used as graft material. We may state that when contemplating tympanoplasty in children with bilateral perforations, tragal cartilage should be considered firstly.

Location and type of perforation have been shownto be prognostic factors influencing surgical outcomes of tympanoplasty. The repair of anterior perforations might be technically difficult especially when the anterior canal is prominent, and blocks accessing the perforation. Some authors [24] proposed that anterior perforations have a tendency to slow and poor healing in tympanoplasty while others [25] found the lowest failure rates in anterior perforations. Some authors [9], on the other hand, did not find any correlation between the location of perforation and graft success. We also did not find a significant correlation between surgical outcome and site of perforation (*p* > 0.05).

Graft success rates were observed to be significantly lower in marginal perforations compared to central perforationsin some studies [5] even though some authors [26] did not find a significant correlation between type of perforation and surgical success. In the present study, even though graft failure was more common in marginal perforations, we did not find any statistically significant relationship between type of perforation and surgical outcome (*p* > 0.05). Marginal perforations have a tendency tolead to cholesteatoma development, which results in ossicular chain erosion and hearing loss, hence it may be stated that a successful tympanoplasty in pediatric patients may prevent the complications associated with a long-term marginal perforation.

Temporalis fascia and tragal cartilage grafts have been most commonly usedin tympanoplasty for many years. Tragal cartilage is strong, thick and easy to place compared to temporal fascia. In a study by Ozbek et al. [27] a higher success rate was observed in the use of tragal cartilage compared to temporal fascia in type 1 pediatric tympanoplasty. In concordance with their results, we found a significantly higher rateof graft success in cartilage group (95.1%) (*p* = 0.033).

In our clinic, postauricular and endaural approaches were performed mostly. Halim et al. [28] compared the success rates of postauricular and endaural approach, and foundno statistically significant difference between two approaches. In this study, 76.5% of operations were performed via the postauricular approach while the rest were performed via the endaural approach. We did not find any correlation between success rate and surgical approach and the findings shows that both two techniques can be performed safely in pediatric patients.

Tympanoplasty is performed to achieve an intact tympanic membrane and hearing improvement. In our study, postoperative ABG significantly decreased compared to preoperative ABG (*p* < 0.001, *p* < 0.001, respectively). Previous studies reported a postoperative mean hearing gain up to 12 dB and a functional success rate ranging from 50 to 88% depending on the definition for improvement [4–6, 10, 12, 25, 27]. Our results were concordant with the literature.

Our study has some limitations. This is a retrospective study and the records of patients obtained from medical charts may be inaccurate or missing. Moreover, only postoperative 6^th^ month hearing results were investigated in our study. However, Future studies with longer follow-up period are needed to support our results.

## Conclusion

In conclusion, we believe that type 1 tympanoplasty can be performed effectively in pediatric population regardless of age, location and type of perforation and surgical approach. Early tympanoplasty has an excellent potential for restoring hearing function and may prevent future complications and deterioration associated with long-term perforations. Bilateral perforations are prone to reperforation, and should be treated with cartilage graft.

## Abbrevations

ABG: Air-Bone gap
dB: Decibel
N.S: Non-significant
PTA: Pure Tone Audiometry
S.D.: Standard deviation
TM: Tympanic membrane
